# Diphtheritic Paralysis

**Published:** 1904-07-30

**Authors:** 


### HOSPITAL CLINICS.

### DIPHTHERITIC PARALYSIS.

THE term diphtheritic paralysis includes cases which differ widely in the degree of their severity, though in all probability they own a common etiological relationship. The most serious and terrible form of the disease is seen in cases of cardiac failure, and it is the liability to this which demands a strict maintenance of the recumbent posture during the progress of the illness. The danger, however, does not cease when the activity of the throat and allied symptoms subsides. Indeed it is perfectly well known that even when convalescence appears fairly well established anything like a sudden increase of cardiac strain may be followed by a serious and perhaps a fatal syncope. Hence the necessity of warning the patient against sudden effort such, for example, as an abrupt resumption of the erect posture. In not a few cases, neglect of this precaution has entailed disastrous consequences. It is of some interest and importance to observe that the liability to cardiac failure may exist in cases which, in their earlier aspects, have been regarded as slight and relatively trivial, and it is at least possible that cardiac failure may be caused by the poison of diphtheria, even though there have been no appreciable local effects in the faucial or neighbouring mucous membranes. Within the last few months a case of rapid failure of the heart in a child, proved on post-mortem examination to be associated with extreme fatty degeneration of the cardiac muscle, has been reported, and the history of the case was entirely free from any incident suggesting diphtheria in its commonly recognised forms. In view of the known tendency of diphtheria to produce such degeneration, it seems reasonable to assume that, in this and parallel instances, the explanation of the cardiac failure was that now suggested. This assumption receives additional support from the fact that the more frequent form of diphtheritic paralysis appears to occur without any relationship to the severity of the initial symptoms. Indeed, it has been held by some that paralysis is more frequent in slight than in severe attacks of faucial diphtheria. In any event it is certain that the throat symptoms may be inconspicuous and possibly altogether overlooked, and yet the subsequent paralysis may be both widespread and severe. It is therefore not difficult to believe that in exceptional instances the most obvious and possibly the only clinical manifestation of diphtheria may be cardiac failure as a consequence of fatty changes in the heart muscle. There is an additional aspect of this tendency of the disease to affect the heart of which it is well a note should be taken. As already indicated, diphtheritic paralysis of the lower limbs and other parts may develop without a decided history of any throat affection, and a hint as to the true nature of the condition may sometimes be obtained from a careful observation of the cardiac rhythm. The disturbance of this may be comparatively slight and may be limited to occasional intervals, but if it exists it is of considerable value for purposes of diagnosis. Another contribution to the same end is obtained if the urine is found to contain albumen. The significance of these two events in cases of paresis of the lower limbs in children is very great, and they are of all the more value when, as is sometimes the case, the paralytic disturbance is slight and more or less indefinite. The importance of these two facts becomes still more obvious when it is remembered that evidences of paresis may not appear for several weeks after the primary symptoms of diphtheria, and that if these have been of a mild order their occurrence may be entirely forgotten.

Concerning the usual manifestations of diphtheritic paralysis, these, it is well known, are usually seen in the form of paralysis of the soft palate, of the ciliary muscle, and of the lower limbs. There are, however, many exceptions to this manner of distribution. Thus the palate may entirely escape. The same is true of the ciliary muscle. And in the case of the lower limbs it is noteworthy that evidence of involvement of the nervous apparatus may be limited to loss of the knee-jerks. If a rule is made to test the knee-jerks in all cases of diphtheria both during and for some weeks after the attack they will be found to be absent in not an inconsiderable number of instances. Walker Downie[1] teaches that absence of the knee-jerks occurs early in diphtheria and may be noted among the first events of the disease. This is certainly not always or even generally the case but the point may be usefully applied when there is difficulty in determining the exact nature of a tonsillar or faucial inflammation. The bacteriological test, valuable as it is, necessarily involves delay, and it has by no means abolished the claim for careful and exact clinical observation with a view to early diagnosis. Another fact of importance in connection with post-diphtheritic effects on the nervo-muscular apparatus is that the most marked abnormality in the lower limbs may be ataxia rather than paralysis. Dr. Chas. R. Box[2] has recorded a very marked example of this condition in which, though muscular weakness was inconsiderable, there was extreme inco-ordination in the movements of all the four limbs. Indeed it may be said that it is frequent in the slighter cases of diphtheritic paralysis to observe an ataxic character of the gait in association with more or less loss of muscular power. Taking it for granted that the cause of the phenomena of diphtheritic paralysis is a multiple neuritis, it may be presumed that when muscular weakness is the leading fact the lesion is mainly confined to efferent (motor) nerves, whilst when ataxia is conspicuous it is afferent (sensory) tracts that are affected. In the latter instance the impressions from the muscles and other parts necessary to secure the adjustment and graduation of the motor impulses discharged from the spinal centres will be cut off, and consequently, with little or no loss of muscular power, attempted movement will be inco-ordinate and disorderly. It has been observed on more than one occasion that the ataxic condition may precede by some weeks loss of the knee-jerks.[3]

In the great majority of cases the sufferers from diphtheritic paralysis are young children. But occasionally adults are affected, and here also the faucial or other early symptoms may be slight, and thus the opportunity for a mistaken diagnosis arises. In a case reported by Dr. Jas. Taylor[4] there was considerable weakness of the lower limbs and inability to see near objects (cycloplegia), but the palate had never been affected—at least, not to such a degree as to produce regurgitation of fluids through the nose or other discomfort. Dr. Dundas Grant[5] has noted an example of diphtheritic paralysis in an adult involving the soft palate, but without loss of the knee-jerks. Speaking generally, it may be said that in adults the prognosis is better even than it is in children. In the latter the prospect is a decidedly good one, though weeks or months may elapse before the complete restoration of function. There is also some risk of cardiac and respiratory paralysis, and hence a period of complete rest is advisable. But in the great majority of cases, rest, massage, electricity, and tonics, such as strychnine, produce the best results, and the patient regains a full measure of muscular power.

A question which has been much discussed is whether non-diphtheritic inflammations of the fauces, palate, and pharynx ever produce a condition simulating diphtheritic paralysis. Probably most physicians would be inclined to say that the occurrence of such a paralysis was a proof of the diphtheritic nature of the throat affection. But there is some evidence which appears to qualify this proposition. It may, indeed, be stated as certain that inflammatory conditions of the throat in which neither the clinical nor the bacteriological characters suggested diphtheria, have been followed by paralyses which corresponded in every respect to those which occur in a certain number of undoubted diphtheritic cases.[6] Mr. Hutchinson[7] dealing with this point quotes examples of cycloplegia as sequels to quinsy and pharyngeal abscess, though he recognises these as quite exceptional clinical sequences. Of equal interest with these records are examples of paralysis, corresponding more or less to the diphtheritic order, after certain other specific diseases. Mumps and pneumonia have each been credited with this responsibility, but the principal interest in this respect is attached perhaps to enteric fever. In a case reported by Dr. Waller G. Stewart,[8] evidences of palatal paralysis and of multiple neuritis affecting all four limbs followed an attack of enteric fever, and Mr. Sydney Stephenson[9] has noted a permanent paralysis of the ciliary muscle in similar circumstances. Experience, therefore, may be advanced in support of the argument from analogy that paralysis of the soft palate though peculiarly liable to be due to the poison of diphtheria may exceptionally be traced to the operation of other specific toxins, and more especially perhaps to the toxin of enteric fever. Some statistics[10] showing the absence of the knee-jerks in 29 out of 100 cases of enteric fever may be held to lend support to this proposition. Paralyses of the external ocular muscles which have been observed to follow measles and other febrile disorders cannot be said to be exactly parallel to diphtheritic paralysis, as in this condition these muscles are very rarely affected. But palatal paralysis, loss of power in the ciliary muscle, and absence of the knee-jerks may be regarded as peculiarly suggestive of diphtheria. It is, therefore, all the more interesting to observe that each one of these has been noted as a sequel of enteric fever.

There is one other consideration, of doctrinal as well as of practical interest, in connection with diphtheritic paralysis on which a single remark may be made. The law which governs its occurrence entirely evades definition. Of two cases equally severe in the degree of faucial disturbance one suffers from paralysis, the other escapes. Or it may be a case slight at the outset is subsequently paralysed, whilst a serious and extreme case passes into complete convalescence without any trace of paralytic development. Such facts are indeed puzzling. That mere chance determines such experiences is a suggestion no one will accept. All the same, satisfactory explanation is, so far, beyond us. Possibly it is to be found less in a variation in the amount or virulence of the poison than in a varying susceptibility of the patient. What exactly this is and upon what circumstances it depends it were hard to say. But in spite of the advance of bacteriology it still remains true that the cause of the symptomatic disturbances which are regarded as the expressions of various diseased states is not simple but complex. And amongst other influences must be included idiosyncrasy, or the capacity for reaction which is to be numbered amongst the factors that determine individuality.

[1] Trans. of the Glasgow Medico-Chirurgical Society, vol. i. [2] St. Thomas's Hospital Reports, vol. xxviii. [3] Buzzard's Harveian Lectures (1888). [4] Polyclinic, vol. vi., p. 132. [5] Ibid., p. 149. [6] Brit. Med. Journ., March 4, 1899, and August 2, 1902. [7] Archives of Surgery, vol. iv., p. 209. [8] Brit. Med. Journ., March 2, 1901. [9] The Ophthalmoscope, January 1904. [10] Brit. Med. Journ., March 23, 1901. [11] Lancet, February 9, 1901.

